# American Heart Month — February 2013

**Published:** 2013-02-15

February is American Heart Month. Heart disease is a major public health problem in the United States. Every year, approximately 715,000 persons in the United States have a heart attack, and approximately 600,000 die from heart disease. Heart disease is the leading cause of death for U.S. men and women, accounting for one out of every four deaths each year (1).

Cardiovascular disease, including heart disease and stroke, costs the United States $312.6 billion each year (1). This total includes the cost of health-care services, medications, and lost productivity. Cardiovascular diseases also are leading causes of disability, preventing affected persons from working and enjoying family activities.

In observance of American Heart Month, CDC will offer four heart-healthy tips per week. This social media and web-based campaign is a way to actively engage persons in heart-healthy activities. Each tip falls into one of four categories: 1) reducing sodium consumption, 2) getting active, 3) quitting smoking, and 4) controlling blood pressure. All the tips will be listed together online at http://www.cdc.gov/salt/healthy_heart_tips.htm and will be accessible all year long.
